# Supplementary figures and images for: Hypoxia-induced tracheal elasticity in vector beetle facilitates the loading of pinewood nematode
Source: eLife. 2023 Mar 30;12:e84621. doi: 10.7554/eLife.84621 (PMC10063229; doi:10.7554/eLife.84621)

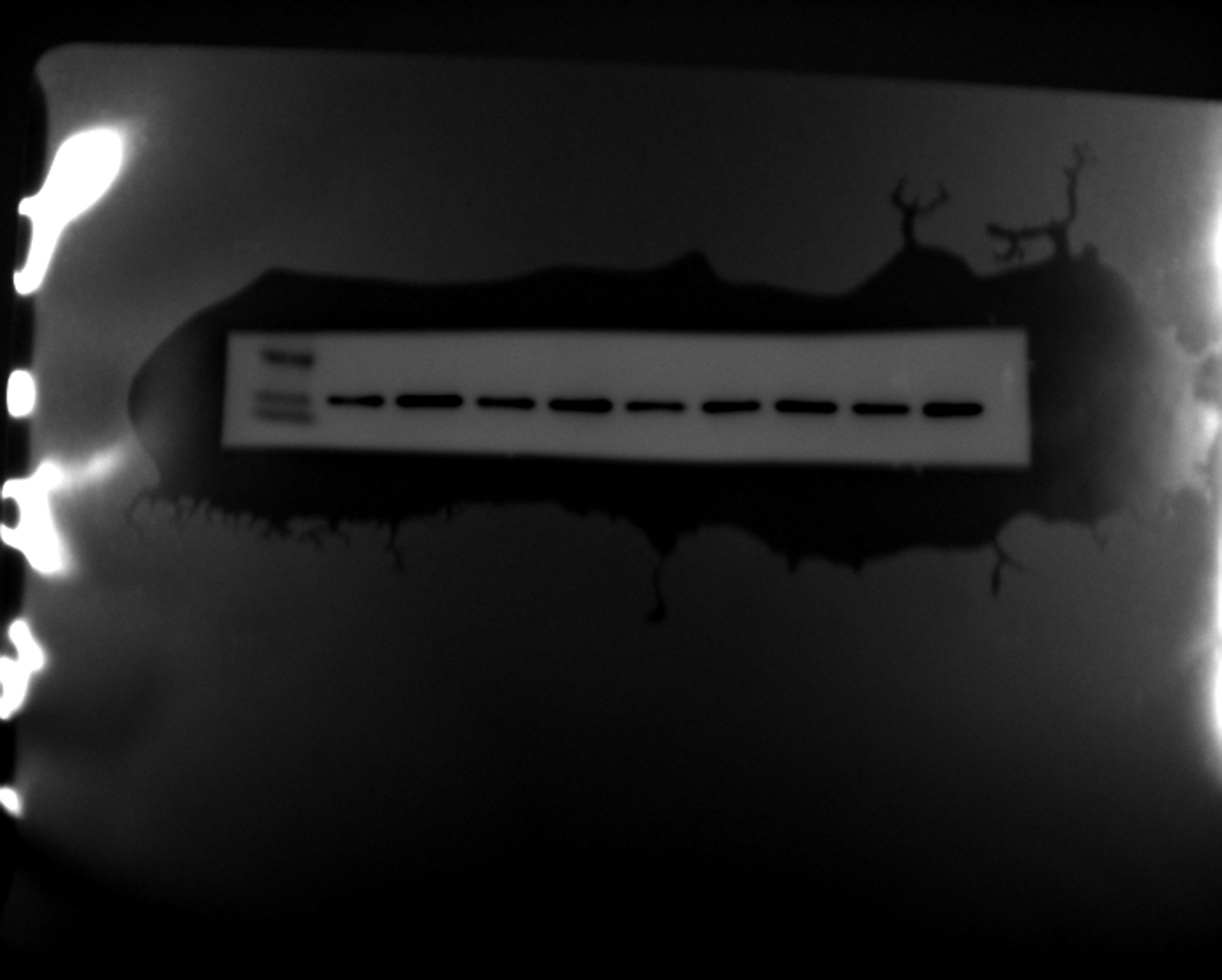

Supplement: Figure 4—source data 2. [file elife-84621-fig4-data2.zip › WB histone h3 merge.Tif]

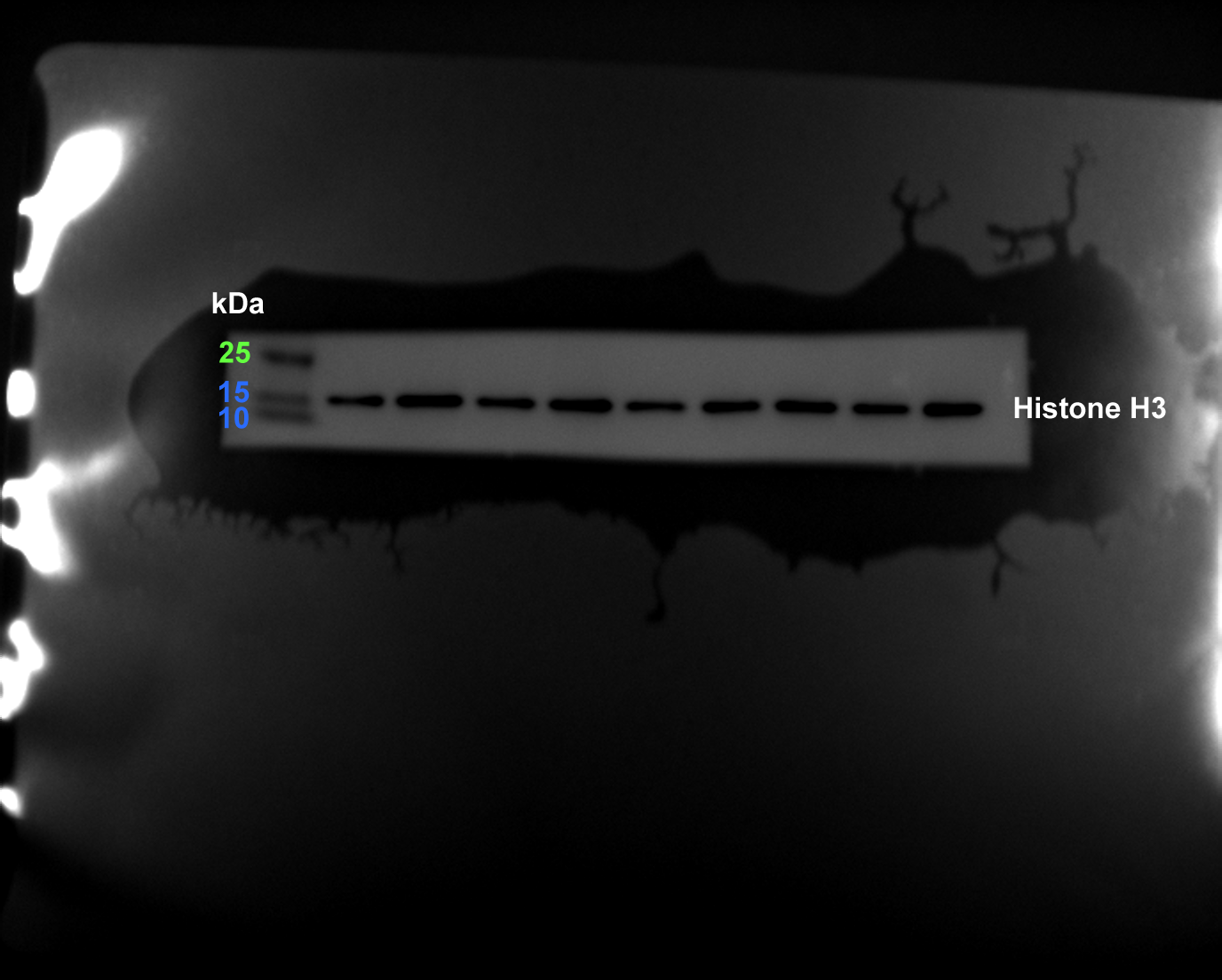

Supplement: Figure 4—source data 2. [file elife-84621-fig4-data2.zip › WB histone h3 merge-labeled.tif]

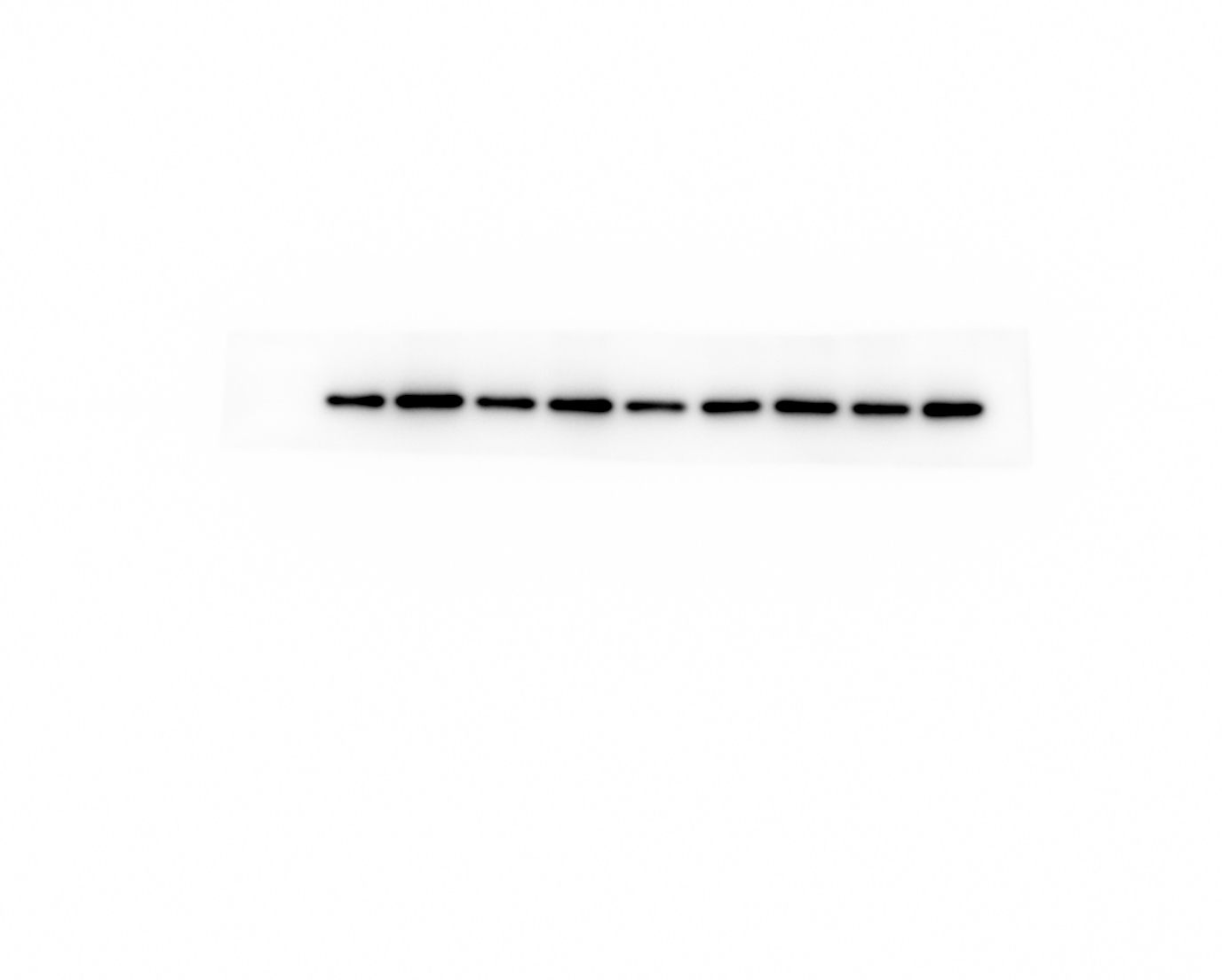

Supplement: Figure 4—source data 2. [file elife-84621-fig4-data2.zip › WB histone h3.Tif]

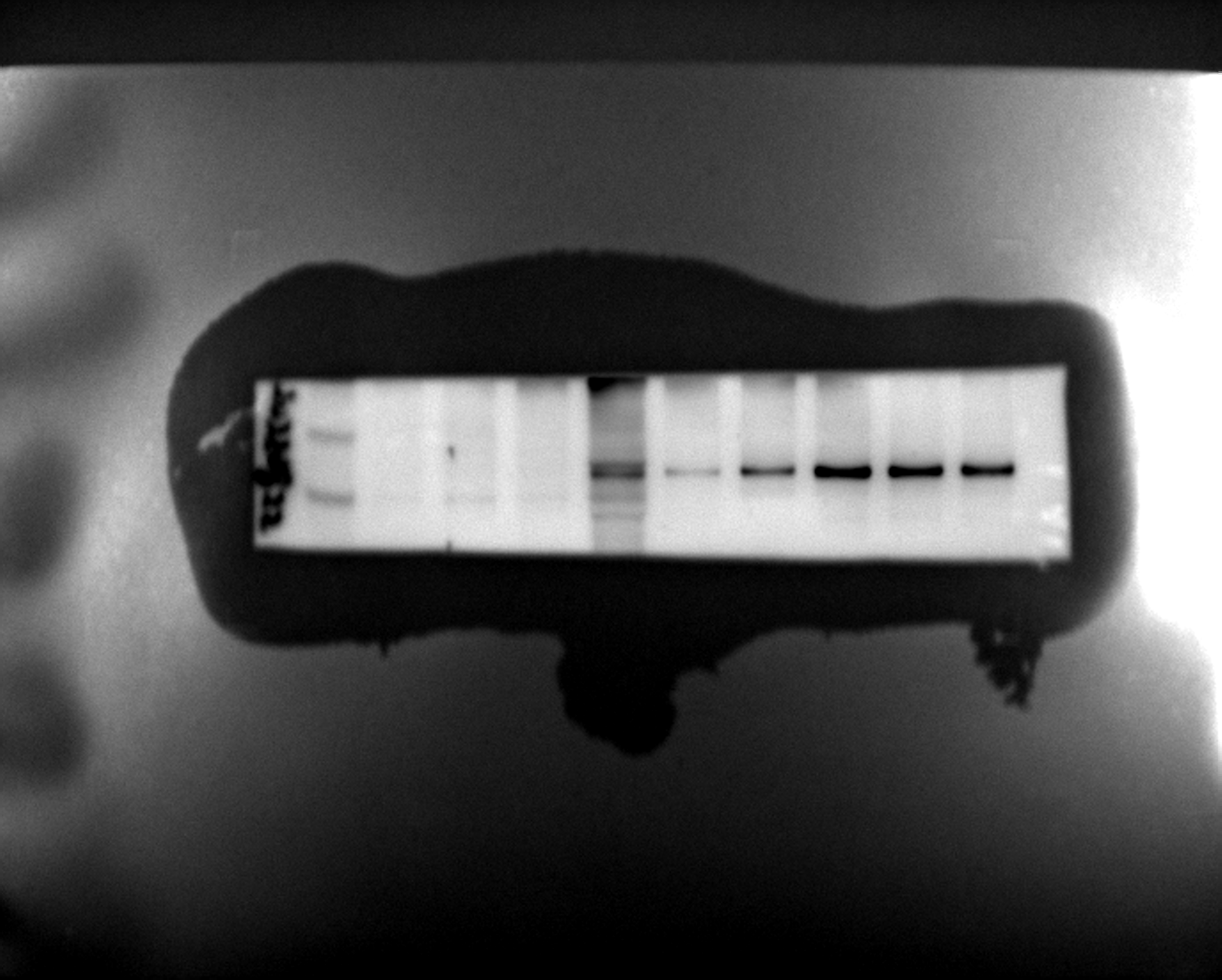

Supplement: Figure 4—source data 2. [file elife-84621-fig4-data2.zip › WB Muc91C merge.Tif]

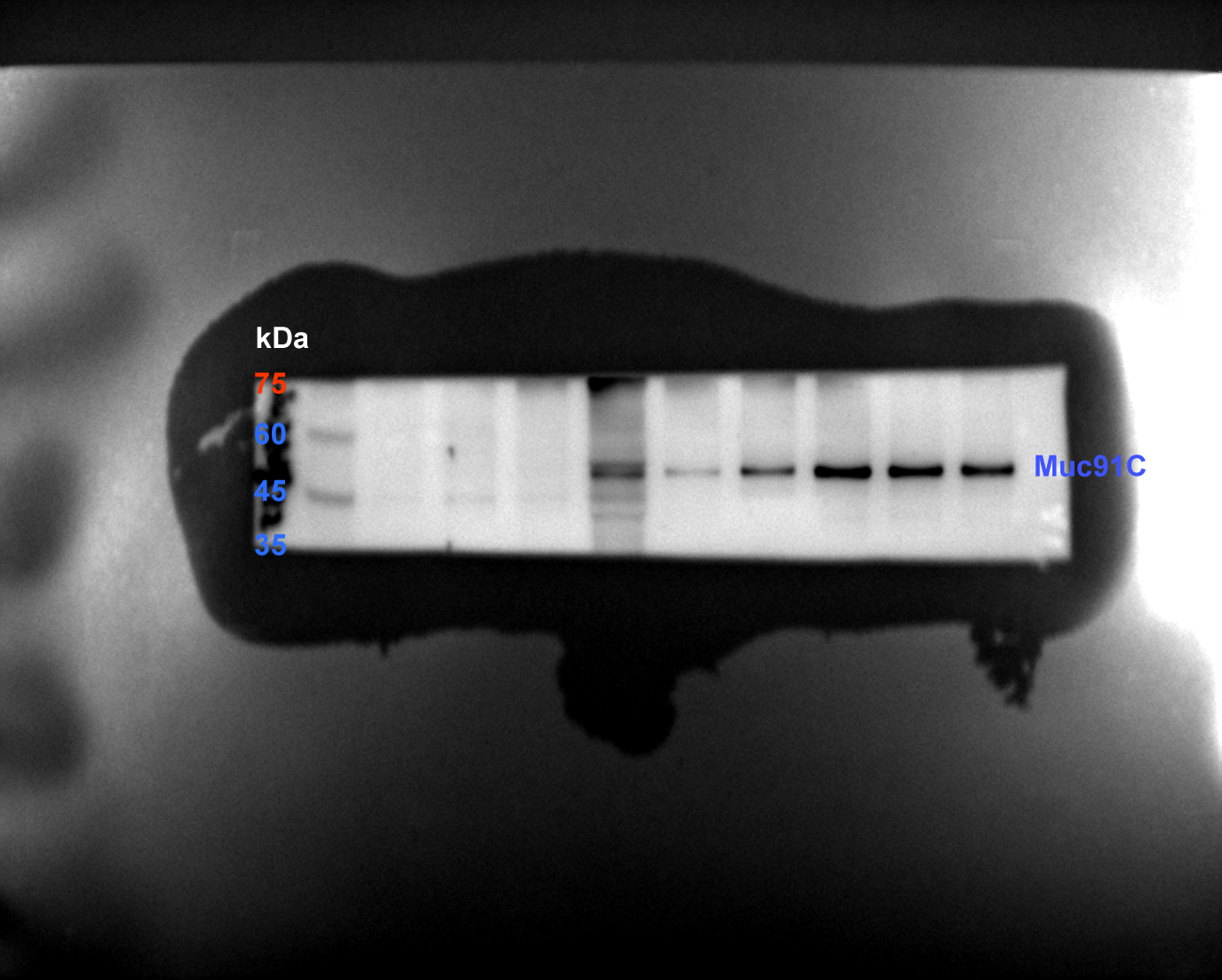

Supplement: Figure 4—source data 2. [file elife-84621-fig4-data2.zip › WB Muc91C merge-labeled.tif]

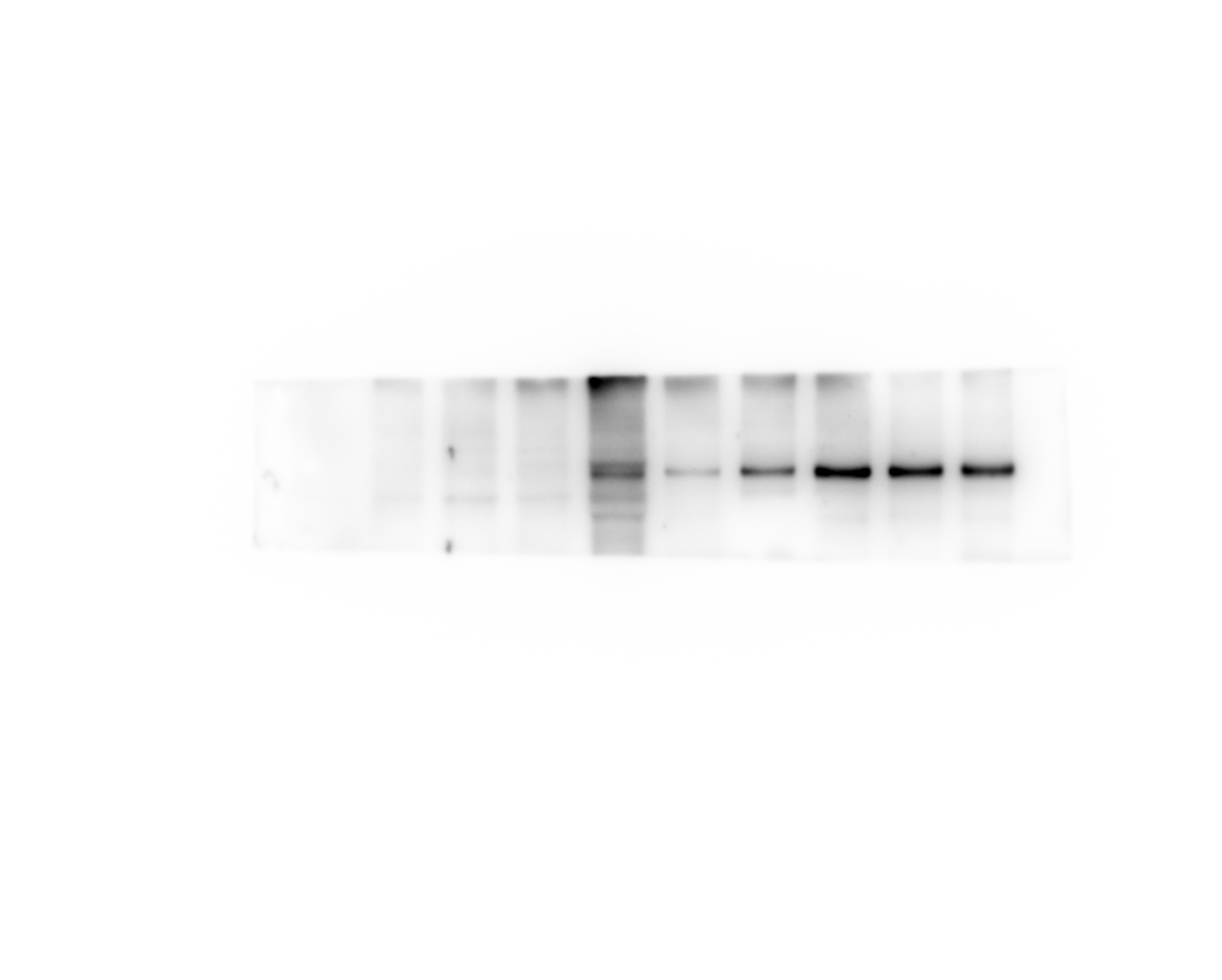

Supplement: Figure 4—source data 2. [file elife-84621-fig4-data2.zip › WB Muc91C.Tif]
