# Supplementary material for: Hypoxia-induced tracheal elasticity in vector beetle facilitates the loading of pinewood nematode
Source: eLife. 2023 Mar 30;12:e84621. doi: 10.7554/eLife.84621 (PMC10063229; doi:10.7554/eLife.84621)
Supplement: Figure 6—source data 1. [file elife-84621-fig6-data1.docx]

>c50691.graph_c0 **mucin 91C**

MSGLQLVALALCCVLSASNAGRLAKRDAPISNGYPSGRPQNTYLPPSPSSSYGAPSNGNGHGGGNGFGGGAPSGSYGAPSAPSSSYGAPSVGGGNGHGGFGGGNGFGGGAPSSSYGAPAAPSSSYGAPAPSSSYGAPSAPSSSYGAPSNGGGFGNGHGSNGGGFGGKGGFGGGAPSSSYGAPAAPSSSYGAPASAPSSSYGAPSNGNGFGRGNGASTSYLPPSTSYGTPVGPSRPSSSYGAPSNGNGNGNGKKPSSSYGAPSNGNGKRPSSSYGAPSRPSSSYGAPSKPSSSYGAPSRPSSSYGAPAPAPSSSYGAPSFGNGGSNGYSSGGNGGYSSGGNGGGYSSGGYSSGGNGGGYSSGGNGGGYSSGGNGGGYSSGGNGGGYSSGSNGGYSSGGNGGYSSGGNGNGGYSSGGGSGGYSNGGGQGYASNGGYSY

>c56000.graph_c0 **mucin-5AC-like**

MANGFIIFLCLTIAASSAFPTAKEADLVTEPKPGDEFVFVQSTDADARPISRKDKVTQQDRPFETSHKVPASLTYSLKDGDAKKPIKPTKPIKQIQKREANPQGAAPAPAGAIKLDIKALLKKYETELKTSTTPKPKVASTTKSGRRRGANKRAKREARREKGVQVTSPSPVLTTAKATPKKVDDDDVPLFGSASEKQRSRIQVKKGPNGQEYEYEYVYYYYDDEEDPKDEKKVTNSHDGPARNQISRGDKSREKPTPEANEVVPSRGSKTRGRQLGEEETVNEERLPANTRFPPRSRNLNTTPIPEEETTKAVTRGRGRGRPTTEATPSSPSASSEADNISDESQGTRGRTRANVRRPSLDLVDSDSFNTHSANSGKPSFPQELPEGPVRFLGATPNERIELDTEEEPEEDEEKSEPKEEEEEEEDDTSPPAEEGTTVMSPMDKVALDLYAISQGTQKLFGEGEDAESSTEGEKIASTEESEVTTEPVTETVEPETTTVTTTTTTTTTTTTTPAPTTTTTTTTTTTEAAPFGRGKFGGGRRGGLGGRKTTAQSAATTEAAPAESKPKGKFGRPSFGGRARPGSKTTAAPAVEEEAHKEEAKPASHSRPSLNRGRFGGASRPRGRTTAATEEPKEESSGSSTAAPSQRPSLPRARPSFNLRTRGRTTAAPAATEDDQPGEESSTSTTEATSSTTAKGRRIAAPGGVRPLRPGPRINLSARGRPGAATTTSTTTTEAPAEDQVAEAEEEHETPDQEASEKEETPAAPVDNSPLGRLRNKNRISVQQRPKAAASAPVQVRRVNPLIARRRPGQTTEAPSTEAPQESAAPEEPEEEDEEGTEAAPSSSTTEEPKGLNKLLAGRRRPGVRTPGTLSHRT

>c56787.graph_c0 **mucin-3A**

MYKRQLSSFVIFWVVLATFHRNVEASPAKRLNRMVREVYPEIEPENEGNQAQVCIVGDLVYGADETVPAEQPCLKCRCQPPGVQCETIQCVKKPGCKAIHKPNKCCPDYQCECEHNGKIYANGEKLETPPGGECKVCYCRGGEVQCAEVSCYIRKDCEGKRVPGKCCPKYDHCPPIDPLPGRSPYTTEINPSNVDKESFEPWSVTRASLNATEDILSPTSLDIQDENKIPDTNYYEVTTPSFKLTEVEDLQKESFLPKITIQEIIPERKEIPITAPPKVELEPQGTLIIEEAEDFLNHSNDLVVTDVDTDSSEISEVFQHPPPILRIGDKLLFLKKGELVPEKDTSTPSSVITIIGAEGLQRGGFEDSGEVHEVNIPKEDEDADLTTNATLVDKIDVLPESNGFISNPEQSLDSNGSASEVNTATSSYPILPLFTTKSTPSTPTTETCTTTSTTDSDIPSSTTESNVLPDVSLQSSSQEISNDTGSTPDSTDISGTTIEALDTLTTEEIKNITEATPVAADEVDNHAEAKELTSASSSTKIYDVVLEQNPAYPPIPDIMTINTDDTSQRFDIDHTEKEFPPYEPSTISPNFKILPDILEIRSNKTVPTNMTHGEWLKLNPDTLINYKAALPDELLKQPAPSDTEDNTEAPNDSNVTETETTTETYTTNEVTEASTSTTEAVTNYSKKLEPTTNTPEIEEERSTESLEESSEEKRESTTKKNSHASIALNENASIENVKEDSSEAEDMSSTKDTVNNSSGEETTPSVLTDPSMIDKEFSDSNQNKTNSDDVEFVDAVKTKKPVIERNIEFIESVNTNKPVRVRNVDPVTSAEPLPKPEKLILPSKIITKRENNPEEGLDVLKQLSKDISAEVTERNLSSEEEERESQEIFKQLLEDTSTSKPKKELNSDEVESVLQRITGVVAQHAIRGQNPGQAILRFLRNQDSRK

>c50691.graph_c0 **mucin 91C**

CACGCATTGTTTTATTTACTTTAATTTTTGCGGCATTCGAATAATTAACAGATTTTACCCACAAGTATCACATCATTAAAATAAGACCTCTATAAAATTACGAGGTCTTGTTCATTAAAATCGGAACATTATTCTATGTTAGGTCACCAGATATTAATTGCTGATCATATTATCAGATGCATTGGATCTAAGGACGACAAAAAGGCTGTTTTTACATAAAAATAATCATCTGTATTTAAATTACAAAATTTTTACAAAAGGTAGGTGTTTAATACTGCAGAAACTAATAAATAAATAAATCTAATTATATACAGTGTTTATCTTGTCGTCTGACTGTACGTTTAGTATGAGTATCCTCCATTGCTGGCATAACCTTGACCTCCCCCATTGCTGTAGCCTCCGCTTCCTCCTCCTGAACTGTACCCTCCATTTCCATTACCTCCTGAGCTGTAACCGCCATTGCCTCCCGAACTGTAACCTCCATTGCTTCCCGAGCTGTATCCGCCACCGTTTCCTCCAGAGCTATATCCGCCACCGTTTCCACCAGAGCTATATCCACCGCCATTTCCACCAGAGCTGTATCCCCCACCATTTCCTCCCGAGCTATATCCACCAGAACTGTAGCCACCACCGTTGCCGCCGGAGCTGTAGCCACCATTTCCACCTGAACTATATCCGTTGGATCCGCCGTTTCCAAAGGAAGGTGCGCCATAGCTGCTTGAAGGAGCTGGTGCTGGGGCACCATAAGAGGAAGATGGTCTGGATGGGGCACCATAAGAAGAAGATGGCTTACTTGGTGCGCCGTAGGAGGAGGATGGCCTACTTGGAGCACCATATGAAGAAGAAGGTCTCTTGCCGTTTCCGTTTGAGGGAGCTCCGTAGGAAGAAGAAGGCTTTTTGCCGTTTCCGTTTCCATTTCCGTTGGATGGAGCACCGTATGAAGATGAGGGGCGGGATGGTCCTACGGGGGTTCCGTAGCTAGTAGAAGGTGGGAGGTAGCTGGTAGACGCTCCATTTCCTCGACCAAAACCGTTACCGTTGGAGGGTGCACCATAGCTGGACGACGGAGCTGAAGCTGGAGCTCCGTAGCTGGACGATGGGGCAGCTGGTGCACCATAGCTGGAGGAAGGGGCGCCACCACCAAAACCACCTTTACCTCCAAAACCTCCTCCGTTTGACCCATGACCGTTGCCGAATCCTCCTCCATTAGAAGGTGCTCCATAGCTAGAGGATGGTGCAGATGGGGCTCCATAACTGGAAGAGGGTGCAGGAGCGCCGTAGCTAGAACTTGGGGCAGCTGGAGCACCATAACTAGACGATGGAGCTCCACCTCCAAAACCATTTCCGCCACCGAATCCTCCATGTCCATTTCCACCTCCTACGGATGGAGCTCCATAACTACTGGATGGTGCAGATGGTGCTCCGTAGCTACCTGAAGGCGCACCACCTCCAAAGCCGTTGCCGCCTCCGTGTCCGTTTCCGTTAGATGGGGCGCCATAAGAGGACGATGGGGATGGGGGAAGGTAGGTGTTCTGGGGGCGGCCTGACGGATACCCATTTGAAATGGGAGCGTCACGCTTAGCAAGTCGTCCGGCATTAGATGCGCTAAGCACGCAGCACAGAGCGAGAGCCACTAACTGCAATCCACTCATGTTCTCACACTTCACTAACTACCACTGGGGGATATTCAAGTCCAGTAGACTTCGATGTCGGCGAAGTCAATGTCTGG

>c56000.graph_c0 **mucin-5AC-like**

ATTGGCACGTGGCAGGCCTGCATTTGCTTAATCCTAAAAACACTTATAAAAGAGTATAAAAAATACAATATGTGAATTTTCTCCTAAGAATAAGGACTAATTTCCTAACCAAGCAATTATTTCAGTAGGAAAACATAAAACAAAGATATTTACAGGTAATAACAGATAATTGAAACGTAACTAATAACCTAAAATTAAATGAAAACTAGGTAACGTCGAGAACTGGTTAAGAATAATAAATAGGCTTAATCTTAACAAGTGTGGACAAATTGTTGATATACTTAATTAACCTACTGAGAACTGCTGAGAAGAATCAATCTTCCTTACGAAATAACAGTTGAGGACGTAATGAGACGTATTTTATTTCTAATAAAATAGCAATTTTGACAGGATCCTACTGGATTTCACAGCACTGGGTAATATGGGATCAATGGAGGCTTCTGAAAAGAGCCCTATAGACGGCAAAAATATATCTATTTATTTTTAGTTGTTAATTTTGCTGCTAAGTTTGATGACACATGTTTAATTTTTCTGCCGTCACGTTTCAAGCCAAACATACGATCACATATACTACATACACACAATCACTTAGTGAAAGAATTCAGTCCTTCAGCCTTAGGTACGATGGGATAGAGTTCCGGGGGTCCTAACGCCTGGCCTCCTCCTTCCGGCTAGTAGCTTATTCAATCCCTTTGGTTCTTCGGTGGTGCTGGAACTCGGAGCTGCTTCCGTGCCTTCTTCATCTTCTTCTTCAGGTTCTTCGGGGGCTGCAGACTCTTGAGGCGCCTCCGTGGAAGGAGCTTCGGTGGTTTGTCCAGGACGTCTCCTAGCTATGAGGGGGTTTACTCTGCGGACCTGGACAGGGGCGCTAGCCGCCGCCTTTGGGCGCTGTTGGACGCTGATGCGATTCTTGTTACGGAGACGACCTAGTGGGCTGTTGTCTACGGGAGCGGCAGGAGTTTCTTCCTTTTCACTTGCTTCTTGATCGGGCGTTTCATGCTCTTCTTCAGCTTCCGCTACTTGATCCTCTGCTGGAGCTTCAGTAGTAGTAGTTGAAGTAGTAGTAGCTGCTCCAGGTCTTCCTCTAGCAGACAAGTTTATTCTTGGTCCAGGTCTGAGCGGCCTGACACCACCAGGTGCAGCTATCCTTCTGCCCTTAGCAGTTGTACTGCTAGTGGCTTCTGTGGTAGAGGTAGAAGATTCTTCACCGGGCTGATCATCTTCAGTAGCAGCAGGTGCGGCAGTGGTGCGTCCTCTGGTTCTTAAATTAAAAGATGGTCGGGCTCTTGGAAGCGACGGACGCTGCGAAGGAGCGGCAGTGCTGCTGCCGGAGGACTCCTCCTTAGGTTCTTCAGTGGCGGCGGTTGTCCGTCCGCGTGGTCTTGAAGCGCCGCCAAAGCGACCTCTGTTCAAAGATGGTCTTGAATGGCTGGCTGGTTTGGCTTCTTCTTTGTGAGCCTCTTCTTCAACAGCTGGTGCAGCGGTGGTCTTGCTTCCAGGTCTGGCTCTGCCTCCGAAACTAGGTCTTCCGAATTTACCCTTTGGTTTGGATTCTGCTGGAGCTGCTTCAGTAGTAGCAGCAGACTGGGCAGTTGTCTTCCTTCCACCAAGACCACCACGTCTTCCACCACCGAATTTACCGCGTCCGAAGGGAGCTGCTTCTGTGGTTGTGGTTGTCGTGGTAGTAGTTGTAGTAGGAGCTGGAGTTGTTGTGGTAGTTGTGGTGGTGGTAGTAGTTGTTGTCACTGTGGTAGTTTCTGGTTCAACAGTTTCTGTTACGGGCTCGGTAGTAACTTCGCTTTCTTCAGTTGAGGCTATCTTTTCGCCTTCAGTACTGCTTTCGGCATCTTCACCTTCGCCGAAGAGTTTTTGGGTTCCCTGAGAGATGGCGTATAGGTCCAAAGCTACTTTATCCATTGGCGACATAACTGTTGTACCTTCTTCAGCGGGTGGGCTTGTATCATCTTCTTCTTCCTCTTCTTCCTCTTTAGGTTCGCTCTTTTCTTCATCCTCTTCAGGTTCTTCTTCTGTGTCGAGTTCGATGCGCTCGTTGGGTGTCGCTCCCAAGAAACGTACCGGTCCTTCAGGGAGTTCTTGTGGAAAAGAGGGCTTCCCAGAGTTGGCACTGTGCGTGTTGAAGCTGTCACTGTCGACCAGGTCCAGGGAGGGCCTGCGGACGTTGGCGCGGGTTCTGCCTCTAGTGCCCTGAGATTCGTCTGAAATATTATCAGCCTCACTGCTTGCGCTGGGACTGCTGGGAGTTGCCTCAGTGGTTGGCCTTCCCCTTCCACGGCCTCTGGTGACTGCTTTGGTTGTTTCCTCTTCCGGAATGGGAGTGGTGTTAAGGTTTCGACTCCTTGGTGGGAACCTGGTGTTGGCCGGAAGCCGTTCTTCGTTGACTGTCTCCTCTTCGCCGAGTTGACGTCCCCTGGTCTTGCTTCCGCGGGATGGAACTACCTCGTTGGCCTCGGGGGTGGGTTTCTCTCGACTTTTGTCGCCTCTGGAGATCTGGTTGCGCGCTGGACCATCGTGTGAGTTGGTAACCTTTTTCTCGTCTTTGGGATCTTCTTCGTCGTCGTAGTAATAATACACGTATTCATACTCGTACTCTTGGCCATTTGGTCCTTTCTTGACTTGAATTCTGGATCTTTGTTTCTCACTGGCAGATCCAAACAGAGGAACATCGTCATCATCGACTTTCTTGGGCGTTGCTTTCGCTGTAGTCAAAACAGGACTGGGACTGGTCACTTGAACTCCCTTCTCTCTTCTGGCTTCTCTTTTTGCTCTTTTGTTGGCGCCGCGACGCCTGCCGGATTTGGTAGTTGAGGCAACTTTTGGTTTCGGTGTGGTGCTAGTTTTTAACTCTGTTTCGTATTTCTTGAGAAGAGCTTTGATGTCCAATTTAATGGCTCCCGCTGGAGCTGGTGCCGCCCCCTGTGGGTTAGCTTCTCTTTTCTGGATCTGCTTTATTGGTTTTGTAGGTTTGATGGGTTTTTTCGCGTCCCCGTCCTTCAGAGAATAAGTCAGAGAAGCAGGAACTTTGTGAGACGTCTCGAAAGGCCTATCTTGCTGGGTGACTTTGTCCTTTCTGGAGATAGGCCTAGCGTCGGCGTCGGTGGACTGGACAAAGACAAACTCATCCCCCGGTTTGGGTTCCGTTACAAGATCCGCTTCCTTGGCGGTGGGAAAAGCGGAGGAAGCGGCTATCGTTAGGCAGAGGAATATGATGAATCCGTTCGCCATGGCAGAGCTTTGCGAGGAAGTCGTCTGGAAATTATATAACTGCGAGCCTGGCAGCAGAAGAAGAAAGAAGTGAGCGAGGGCGGCAGTATCGCCGCTAAGAGCCGCACTGGGAACAACACTGTGTCACTGTGGAAGTCTCACCTTACACTCAATCACAACGCCACATGTCCGAGACTGCACAATCGGTAACTTCGCGGGGCAACTTTCGCTGTCTTGCGGCCGAGAGTAGTCGGCCTACCACCGTCCGCCCGCCTTTTATATGTCAACTCTTACCCCGTGTCCTGGCCGCAGAGTTTTGCAAAACCGCGTGTTTTTTGTGAGTCGGACAGTGGATGCTTTTGTTGTTTTGGGAAATATTCGATGAATGCGAGACCTGAAGTACCAACTAGTACCCTGAG

>c56787.graph_c0 **mucin-3A**

CACGTTGACTCAACACACAGTCGGTAGGAAGGCCGGTCGACGCACGAGATTCGAAAGTGCAGCAGCGAGCTGCCGATCGACTCGATTCAATTCGAACCGTCAACGACTCGAACTTCGCGGTTCCCTAACGCACCGACATCGATTTAACAACTCGATTGCCGCCGTATTTTGTGTGCCAATGTGCGATTGAGATGTGACATTCTGGACATACTCAGGTATTGTGCGCGAGATTTTGGTTCAGCGGTGCTTGTGCAGATCCGCAAGAGAAGAAGTGAGATGCCATAAACTTTTTCGACCGCCGAAGCCTATTGTACAACATAGTAACGATATATACAGATTTCAAGATGTACAAACGACAGCTGTCAAGCTTTGTGATATTCTGGGTGGTATTAGCAACTTTTCATAGGAATGTGGAAGCGAGTCCTGCGAAACGGCTGAACCGAATGGTGAGGGAAGTGTACCCGGAAATAGAGCCGGAAAATGAAGGAAATCAGGCGCAGGTGTGCATCGTTGGAGACCTGGTGTATGGAGCCGATGAGACCGTGCCAGCCGAGCAGCCCTGCTTAAAATGCAGATGCCAGCCGCCAGGAGTGCAATGCGAGACTATACAGTGCGTGAAGAAACCCGGGTGCAAAGCCATCCACAAGCCTAACAAGTGCTGTCCCGACTACCAATGCGAATGCGAGCATAACGGAAAAATTTATGCAAATGGAGAAAAGCTTGAAACGCCACCTGGAGGAGAGTGCAAGGTCTGTTATTGCAGAGGAGGAGAGGTACAATGCGCTGAGGTGTCATGTTACATAAGGAAAGATTGCGAAGGGAAAAGGGTACCAGGAAAATGCTGCCCCAAATATGACCACTGCCCTCCAATAGATCCACTTCCGGGCAGGTCTCCATATACGACGGAAATAAACCCGTCGAACGTAGATAAGGAAAGTTTCGAACCCTGGTCCGTTACACGAGCTTCATTGAACGCTACCGAAGACATCTTGAGCCCGACGTCCCTCGATATCCAGGACGAAAACAAAATTCCAGACACAAATTACTACGAAGTAACGACACCTAGCTTCAAGTTAACAGAAGTGGAAGATCTTCAAAAGGAATCGTTCCTTCCCAAAATAACCATTCAAGAAATCATTCCAGAGCGTAAGGAAATACCAATAACTGCTCCTCCTAAGGTGGAGCTTGAACCGCAAGGCACTTTGATTATAGAAGAAGCCGAAGATTTCCTGAATCACTCCAATGATTTGGTTGTTACGGATGTTGACACAGATTCAAGCGAGATATCCGAAGTCTTCCAACATCCTCCTCCGATTTTAAGGATTGGAGATAAACTGTTGTTCCTCAAGAAAGGGGAACTTGTGCCTGAGAAGGATACAAGCACTCCCAGCTCCGTGATAACTATAATAGGAGCAGAAGGGTTGCAACGAGGGGGTTTTGAAGATAGCGGAGAAGTACACGAAGTGAACATTCCAAAGGAAGATGAAGATGCGGATCTTACCACCAACGCAACTTTAGTTGACAAAATCGACGTACTGCCTGAAAGTAATGGCTTTATAAGTAACCCAGAGCAAAGTTTAGATTCCAATGGTAGCGCCTCGGAAGTGAACACAGCAACATCTTCTTACCCGATTTTACCTTTGTTTACAACAAAAAGTACGCCCTCTACTCCCACGACGGAGACTTGCACAACTACCTCCACCACGGACTCTGATATTCCAAGTTCCACAACAGAATCCAATGTTCTGCCGGACGTTTCCCTTCAAAGTTCTAGTCAAGAAATATCCAATGACACGGGTAGTACACCCGATAGCACCGATATATCAGGAACCACCATAGAAGCTCTAGACACTCTTACTACTGAGGAAATAAAGAACATTACAGAAGCAACACCTGTAGCAGCAGATGAAGTAGATAATCATGCAGAAGCAAAAGAATTAACTTCAGCTTCGAGCAGCACAAAAATCTACGACGTGGTGTTGGAACAAAATCCAGCATATCCTCCTATTCCGGACATAATGACTATAAATACTGACGACACCTCTCAGAGATTCGACATAGATCACACCGAAAAAGAATTTCCACCTTATGAACCCTCGACTATATCCCCCAACTTCAAAATTCTACCAGATATATTGGAAATAAGAAGTAACAAAACGGTGCCAACAAATATGACGCACGGAGAATGGTTGAAATTAAACCCAGACACTTTGATCAACTATAAGGCTGCTCTACCCGACGAATTACTCAAACAACCTGCTCCAAGTGACACCGAAGATAATACAGAAGCTCCCAACGATAGTAATGTTACAGAAACAGAAACGACGACTGAAACCTACACCACGAATGAAGTTACAGAAGCTAGTACTAGTACAACAGAAGCGGTGACAAATTACAGCAAAAAGTTGGAACCAACCACAAACACTCCAGAAATAGAAGAAGAAAGGTCGACTGAAAGCTTGGAAGAATCAAGTGAAGAGAAAAGAGAATCCACAACAAAAAAGAACAGCCACGCCTCGATAGCACTCAATGAAAATGCAAGCATTGAAAATGTCAAAGAAGACTCTTCAGAGGCTGAGGATATGTCGTCAACCAAAGACACAGTTAACAATAGCAGCGGTGAAGAAACTACACCATCCGTTTTAACGGATCCCAGTATGATAGACAAGGAATTCAGCGATTCAAACCAAAACAAAACCAACAGCGACGATGTCGAATTCGTGGACGCCGTAAAAACGAAGAAACCCGTAATTGAAAGAAATATTGAATTTATAGAGAGTGTAAACACAAATAAACCTGTCAGAGTAAGGAACGTGGATCCTGTTACCTCTGCGGAGCCTCTACCAAAGCCAGAAAAGCTTATTTTACCCTCCAAAATCATCACGAAGAGAGAAAATAATCCTGAAGAGGGATTAGACGTTTTGAAACAACTCAGTAAGGATATAAGCGCCGAGGTGACAGAAAGGAATCTGTCTAGTGAGGAAGAAGAGAGGGAATCTCAGGAGATTTTCAAGCAACTCTTAGAGGACACAAGTACCTCTAAACCGAAAAAAGAACTCAACAGCGATGAAGTTGAATCGGTTCTACAGAGAATTACGGGCGTGGTTGCTCAACACGCAATAAGAGGACAAAATCCTGGTCAAGCAATACTCAGATTTTTAAGAAATCAAGACAGTAGAAAATAGGCTCGTACATTTCTCGAATAGAAAAGGATTTGTTTGGGAACGAGATTGAAGACAGAAATGGATGGATGTGGACGTTGGATAAAAACGTTATTAGTGTGATTTTGCCAATATTGCTGAGACTTTATTTATGAATGTTCGCTTAGGACATACACTTTATGTTTCTAGAAATAAACTGTAACGTTTAATTAGGATAAGTGTAATGACCCTTAAAAGTATTCCATACTAGTACTACTGTGTAGGATCATAATTTATTTATTATTTAGATTATAGATAGAGCGAAGACAAAATTTATTTATAACTGAAATTTTATTATTCCTATCATTGTTACTAGTTGATTATATATGTAACTACATGTAACAGA
