# Supplementary material for: Hypoxia-induced tracheal elasticity in vector beetle facilitates the loading of pinewood nematode
Source: eLife. 2023 Mar 30;12:e84621. doi: 10.7554/eLife.84621 (PMC10063229; doi:10.7554/eLife.84621)
Supplement: Supplementary file 1. [file elife-84621-supp1.docx]

| **Supplementary file 1**. Species and GenBank accession no. of Pro-resilins and mucins | | |
| --- | --- | --- |
| Species | Protein | Acession Number |
| *Anopheles gambiae* | Muc91C | AGAP002367 |
| *Anoplophora glabripennis* | Muc91C | XP_018561641.1 |
|  | Pro-resilin | XP_018561641.1 |
|  | Muc3Al | XP_018564600.1 |
|  | Muc3A | XP_018562266.1 |
|  | Muc2 | XP_018564844.1 |
|  | Muc3Al | XP_018564600.1 |
|  | Muc5AC | XP_018564744.1 |
| *Acyrthosiphon pisum* | Muc2 | XP_008181271.1 |
| *Aedes aegypti* | Muc3A | XP_021708482.1 |
| *Apis mellifera* | Muc91C | XP_006569520.2 |
| *Bombus impatiens* | Muc3A | XP_012243416.1 |
|  | Muc5AC | XP_012239483.1 |
| *Bombyx mori* | Muc3A | XP_037870848.1 |
| *Drosophila melanogaster* | Muc91C | AAF55584.1 |
|  | Pro-resilin | NP_611157.1 |
| *Drosophila willistoni* | Muc5AC | XP_023031088.1 |
| *Homo sapiens* | Muc5AC | NP_001291288.1 |
|  | Muc2 | AZL49145 |
|  | Muc3A | XP_021708482.1 |
| *Myzus persicae* | Muc91C | XP_000059040.1 |
|  | Pro-resilin | XP_000004150.2 |
| *Nasonia vitripennis* | Muc91C | XP_001605137.1 |
|  | Pro-resilin | XP_001604687.1 |
| *Papilio xuthus* | Muc2 | XP_013171518.1 |
| *Tribolium castaneum* | Muc91C | XP_975482.1 |
|  | Muc5AC | XP_008198760.1 |
|  | Muc2 | XP_015834638.1 |
|  | Pro-resilin | XP_001807028.1 |
|  | Pro-resilin | XP_006563165.2 |
| *Zootermopsis nevadensis* | Muc5AC | XP_021921012.1 |
|  | Muc3A | XP_021933252.1 |
|  | Muc2l | XP_021912795.1 |
