# Supplementary material for: Hypoxia-induced tracheal elasticity in vector beetle facilitates the loading of pinewood nematode
Source: eLife. 2023 Mar 30;12:e84621. doi: 10.7554/eLife.84621 (PMC10063229; doi:10.7554/eLife.84621)
Supplement: Supplementary file 2. [file elife-84621-supp2.docx]

**Supplementary file 2**. Primers for real-time qPCR and RNAi experiments

|  | Primer, 5‘–3’ | |
| --- | --- | --- |
| Gene | sense | antisense |
| Muc91C | AATGGAGGTTACAGTTCGG | TTGCTGGCATAACCTTGA |
| Muc5ACl | CTCCCGTAGACAACAGCC | TTCCGTGCCTTCTTCATC |
| Muc3A | TGAACCGAATGGTGAGGG | GCTTGTGGATGGCTTTGC |
| RP49 | AAGGCGTTTCAAGGGACA | GGGCACGTTCTACGATTTCT |
| *ds*GFP | TAATACGACTCACTATAGG  AACGGTTCAAAGCTTCCGAC | TAATACGACTCACTATAGG  ATAGCGGTTCGTTCCAATGC |
| *ds*Muc91C | TAATACGACTCACTATAGG  CAAATGGGTATCCGTCAGG | TAATACGACTCACTATAGG  GAGCACCGTATGAAGATGAG |
| Red font indicates T7 promoter sequence. | |  |
